# Evolution of Problematic Mobile Phone Use in the Spanish Population over the Last Decade

**DOI:** 10.3390/bs16010008

**Published:** 2025-12-19

**Authors:** Jose de-Sola, Joan I. Mestre-Pintó, Victor J. Villanueva-Blasco, Hernán Talledo, Antonia Serrano, Gabriel Rubio, Fernando Rodríguez de Fonseca

**Affiliations:** 1 Grupo de Neuropsicofarmacología, Instituto de Investigación Biomédica de Málaga y Plataforma en Nanomedicina-IBIMA Plataforma BIONAND, Unidades Clínicas de Neurología y Psiquiatría, Avenida Severo Ochoa 35, 29590 Málaga, Spain; jose.desola@desaludpsicologos.es (J.d.-S.); antonia.serrano@ibima.eu (A.S.); 2 De Salud Psicólogos, Centro de Psicología y Psicoterapia, Dr. Castelo 42, 28009 Madrid, Spain; 3 Addiction Research Group (GRAd), Neuroscience Research Program, Hospital del Mar Research Institute, 08003 Barcelona, Spain; jmestre@researchmar.net; 4 Faculty of Health Sciences, Valencian International University, Pintor Sorolla 21, 46002 Valencia, Spain; vjvillanueva@universidadviu.com; 5 Department of Marketing and Communication, Universidad Peruana de Ciencias Aplicadas, Lima 150140, Peru; pccmotal@ucp.edu.pe; 6 Departamento de Psiquiatria, Universidad Complutense de Madrid, Instituto de Investigación i+12, Hospital Universitario 12 de Octubre de Madrid, 28041 Madrid, Spain; grubiovalladolid@gmail.com

**Keywords:** cell phone, problematic use, dependence, craving, anxiety, videogaming, prevalence, Mobile Phone Problematic Use Scale (MPPUS), Mobile Phone Addiction Craving Scale (MPACS)

## Abstract

This study assessed problematic mobile phone use in the Spanish population between 2014 and 2025 using the Mobile Phone Problematic Use Scale (MPPUS) and the Mobile Phone Addiction Craving Scale (MPACS). Two online surveys were conducted in 2018 (n = 1612) and 2024 (n = 2001) across Spain’s 17 autonomous communities, with analyses by gender, age, occupation, education level, and population size. Data were compared with a 2014 baseline study (n = 1126). The prevalence of problematic mobile phone use declined slightly from 5.1% in 2014 to 4.8% in 2018 and 3.2% in 2024. Users reporting difficulties controlling their phone use (problematic and at-risk users) also decreased from 20.5% in 2014 to 18.8% in 2024. However, the severity of problematic use increased over the decade, as reflected by rising MPACS scores among problematic users. Major factors associated with problematic use included hours of daily use, age (especially under 35 years), anxiety symptoms, and videogaming, while gender and alcohol consumption showed minor influence. Despite the slight reduction in prevalence, the growing intensity of problematic use highlights its persistence as a public health concern among young adults, underscoring the need for preventive and therapeutic interventions.

## 1. Introduction

The use and abuse of mobile phones, as well as the resulting problems, are no longer novel phenomena. Since the first academic studies raised concerns more than 20 years ago, continuous warnings have been issued in the media about the dangers of indiscriminate mobile phone use. As this line of research has expanded, new mobile devices such as tablets have emerged, shifting attention away from Internet addiction on home computers. Regarding mobile devices, many developments have occurred since the earliest studies. Initially, research focused on the devices themselves, later evolving to examine specific applications such as social networks, music, photography, or video games. With the advent of smartphones, the role of applications in uncontrolled and problematic use became evident (De-Sola et al., 2019), giving rise to two main lines of research: (1) the mobile phone as a constantly available tool for distraction, entertainment, and relief of restlessness or boredom, and (2) specific applications—such as social networks (Andreassen, 2015; Andreassen et al., 2012; Karaiskos et al., 2010), WhatsApp (Alkhalaf et al., 2018), video games (Fransson et al., 2018), or photography (“selfies”) (Starcevic et al., 2018)—that satisfy needs for sharing, visibility, and self-presentation.

Although early studies emphasized the risks of mobile phone abuse among adolescents and young people, subsequent research has shown its progressive spread to the adult population (De-Sola et al., 2017a; Montag et al., 2015; Smetaniuk, 2014). Thus, problematic mobile phone use has not only become entrenched but now represents a source of difficulties across all age groups. The technology embedded in mobile devices is inherently attractive, rendering them potentially addictive—particularly when used to distract oneself, alleviate boredom or anxiety (Leung, 2007), maintain highly dependent social relationships (Cha & Seo, 2018), enhance visibility and social belonging, seek novelty (Leung, 2007), or avoid loneliness. These factors may explain the sense of urgency and anxiety experienced when the device is unavailable (Leung, 2008; Park & Lee, 2011; Salehan & Negahban, 2013).

Since the earliest studies on problematic mobile phone use, there has been debate over whether such behaviors constitute true addiction or merely problematic use. Manifestations associated with mobile device use share diagnostic features with substance addictions and with other behavioral addictions—such as compulsive shopping, eating, sexual behavior (Otero-López et al., 2011; Phillips et al., 2015; Pivarunas & Conner, 2015; Young, 2008), or gambling—recognized in the DSM-5. Although not yet formally included in major diagnostic classifications (DSM or ICD), many scholars have long considered excessive mobile phone use an addiction. Clinical evidence supports this perspective, as cases of *nomophobia* (fear of being without a phone due to forgetfulness, lack of signal, battery depletion, or malfunction) (Argumosa-Villar et al., 2017); *FOMO* (fear of missing out) (Fuster et al., 2017); and *Hikikomori syndrome* (Koyama et al., 2010; Teo, 2010)—voluntary social withdrawal maintained through digital communication—exhibit dependence and craving when device use is restricted (De-Sola et al., 2017b).

From a theoretical perspective, problematic mobile phone use can be conceptualized within Griffiths’ components model of addiction, which posits that all addictions share six core features: salience, mood modification, tolerance, withdrawal, conflict, and relapse (Griffiths, 2005). These components have been widely applied to behavioral addictions, and instruments assessing problematic technology use (e.g., social media or smartphone use) often operationalize them when defining addictive (Billieux et al., 2015; Fournier et al., 2023). In addition, the I-PACE model (Interaction of Person–Affect–Cognition–Execution) proposes that predisposing variables (e.g., personality traits, psychopathology), affective and cognitive responses (e.g., craving, expectancies, coping motives), and executive functions interact over time to foster the development and maintenance of specific Internet-use and other behavioral addictions (Brand et al., 2019). Longitudinal evidence in children and adolescents supports this framework, showing that problematic Internet use and emotional variables such as depression, anxiety, and emotional symptoms are dynamically related over time (Villanueva-Blasco et al., 2026), underlining the central role of affective processes in digital behavioral addictions. Within this perspective, the Mobile Phone Problem Use Scale (MPPUS) can be understood as an indicator of overall severity of problematic mobile phone use, capturing dimensions such as loss of control, withdrawal symptoms, and interference with daily life (Bianchi & Phillips, 2005; De-Sola et al., 2017c), whereas the Mobile Phone Addiction Craving Scale (MPACS) was explicitly developed to assess craving for mobile phone use in adult populations (De-Sola et al., 2017b), thereby tapping affective-cognitive processes, particularly cue-reactivity and craving, that closely correspond to the mechanisms emphasized in the I-PACE model (Brand et al., 2019).

The present study analyzes the prevalence of problematic mobile phone use, associated factors, and related craving among the Spanish population from 2014 through 2024. Our 2014 findings revealed an overall prevalence of 20.5% of mobile phone use problems, with 15.4% showing abusive or risky behaviors and 5.1% displaying dependency and loss of self-control, comparable to addiction. Craving measured by the MPACS was closely related to the level of problematic use (De-Sola et al., 2017a, 2017b), as well as to factors such as age, anxiety, impulsivity, and alcohol consumption (De-Sola et al., 2017c). Here, we present sequential analyses from 2018 and 2024 using the same methodology. Given the large, nationally representative samples covering 17 of Spain’s 19 autonomous communities, data were analyzed both globally and by sociodemographic variables (age, gender, education, occupation, and population size). The 2024 survey additionally assessed psychological variables, including the impact of videogaming. Our findings indicate that mobile device use has become widespread throughout society and that, despite a modest decline in prevalence, the severity of dependence has increased over the past decade.

## 2. Materials and Methods

### 2.1. Procedures and Instruments

The research was carried out via online surveys designed by our research group (De-Sola et al., 2019, 2017a, 2017b, 2017c) and conducted by a sociological research company (ODEC S.L.) between May and June of 2018 and 2024. Before administering the survey, we conducted a pilot study to evaluate the clarity and usability of the general questionnaire. Seventeen volunteer participants completed the full set of instruments. Their data was used exclusively to refine the general sociodemographic and mobile phone use habits set of questions and were not included in the final sample. The adjustments concerned only this general set of questions and involved minor re-wording of items whose phrasing participants reported as unclear, small modifications to the response format, and the clarification of several terms to ensure consistent interpretation. No items were removed or added. The specific validated instruments (Spanish-adapted versions of the MPPUS, BDI-21, STAI-S, IGDS9-SF, etc.) were not altered, as their official Spanish validations had been performed previously by other authors. The pilot study also allowed us to estimate the average time needed to complete the entire battery and to adjust the e-survey settings accordingly. The final version of the general sociodemographic and mobile phone use habits questions is provided in the Supplementary Materials section. The participants were sent an email invitation with a link to access the interview, which could be completed, with interruptions, in as many sessions as necessary. Once the questionnaire had been completed, the link became unusable, making it impossible to reuse. The extraction and selection of the sample was performed using a database, with a total of 472,922 active collaborators in Spain, owned by the sociological research companies. The use of this type of database is common and frequent in sociological and market studies and is composed of individuals who, voluntarily or in exchange for a reward, participate in this type of research. In our case, the selection of our sample was performed following criteria that accounted for population balance and representativeness in relation to age, gender, main job or occupation, education level and population center type (rural or urban) as variables of analysis as well as geographic distribution in 17 of the 19 Spanish autonomous communities. Furthermore, all participants had to have, at least, their own mobile phone, an aspect that was evaluated through a filter question that conditioned the continuation of the questionnaire. In addition, several scales associated with factors identified in 2014 as relevant contributors to problematic phone use were included, Thus, the questionnaire contained the following instruments: a set of sociodemographic questions, the Mobile phone problematic use scale (MPPUS, (Bianchi & Phillips, 2005; De-Sola et al., 2017c)) and the mobile phone addiction scale (MPACS, (De-Sola et al., 2017b)). In addition, in the 2024 survey we added the Alcohol Use Disorders Identification Test (AUDIT), the Beck Depression Inventory (BDI-21), and the State Anxiety Inventory Scale (STAI-S) as described previously (De-Sola et al., 2019, 2017a, 2017b, 2017c) as well as the Internet Gaming Disorder Scale–Short Form (IGDS9-SF, (Pontes et al., 2017)).

### 2.2. Ethics Statement

The study and recruitment protocols were approved by the Ethics Committee of the Hospital Regional Universitario de Málaga (Code FRF1, last approval: 1 September 2022) and were conducted in accordance with the Declaration of Helsinki (seventh revision, 2013, Fortaleza, Brazil). The internet-based survey included a specific informed consent form that provided a clear description of the nature and objectives of the research. Participants were required to sign the informed consent electronically to gain access to the questionnaire. No personal identifying information (e.g., name, address) was collected in order to preserve the anonymity of the respondents.

### 2.3. Sample and Participants

The sample consisted of 1612 participants in 2018 and 2001 participants in 2024 (men and women aged 16 years and older) from across Spain. The sample distribution was proportional to the population size in 17 of the 19 Spanish autonomous communities, according to data from the National Institute of Statistics (Instituto Nacional de Estadística, INE, 2017). The two autonomous cities, Ceuta and Melilla, were excluded due to their small population size and limited representativeness. Table 1 presents the main characteristics of the participants. Most respondents lived in urban areas (over 50,000 inhabitants), while the remainder resided in rural areas or small population centers. Regarding age and gender, participant selection was determined by quotas to ensure equal representation of men and women. Given the sustained presence of problematic users in the 16–35-year age group in the 2014 and 2018 samples, the proportion of participants in this age range was increased in 2024. With respect to primary occupation, more than half of the participants were engaged in paid employment, while the rest were students, homemakers, unemployed individuals, or retirees. In terms of educational level, over half had completed higher education, about 40% had secondary education, and approximately 7–8% had only basic education or no formal schooling.

### 2.4. Statistical Analysis

This research includes an analysis and review of both, the MPPUS (Mobile Phone Problematic Use Scale) (Bianchi & Phillips, 2005; De-Sola et al., 2017c) for the evaluation of problematic cell phone use and the MPACS (Mobile Phone Addiction Craving Scale) for the assessment of craving performed prior to the surveys of 2018 and 2024. In the case of the MPPUS, we carried out a new review of the adaptation performed in 2005, while for the MPACS, the stability of the instrument was reviewed without any final changes being made (De-Sola et al., 2017a, 2017b). In both cases, internal consistency was analyzed using Cronbach’s alpha, and internal validity was analyzed using factorial principal component analysis. Likewise, Pearson correlations were obtained between the MPPUS and the MPACS for the assessment of external validity, as were statistical overlaps with frequencies and means in the analysis of the prevalence in relation to the sociodemographic variables considered. The maximum statistical significance criterion in all cases was 5%. In the categorization of mobile phone users, the four categories of the MPPUS (casual, regular, at-risk and problematic users) used in previous research (De-Sola et al., 2017a) and proposal by López-Fernández et al. (2012) have been maintained. Additionally, in this research, to improve the analysis of global prevalence, we created the category ‘Users with difficulties’, i.e., the sum of at-risk and problematic users.

Significance of differences in qualitative and quantitative variables was determined by Fisher’s exact test (Chi-square), Student’s T test or and Mann–Whitney U test, as required. Post hoc tests for multiple comparisons were performed using ANOVA followed by the Bonferroni correction test. Correlation analyses were performed using the Spearman’s coefficient (rho). The normal distribution of the variables was assessed using Lilliefors corrected Kolmogorov–Smirnov test. Binary logistic regression analysis for analyzing factors predicting problematic mobile phone use was performed using Pearson’s Chi-square (χ^2^) test with the Hosmer–Lemeshow test. Statistical analyses were carried out using GraphPad Prism version 5.04 and IBM SPSS Statistic version 22 (IBM, Armonk, NY, USA). A *p* value < 0.05 was considered statistically significant.

## 3. Results

### 3.1. Update of MPPUS and MPACS Scales

The MPPUS was originally designed and validated by Bianchi and Phillips (2005) in a sample of individuals aged 18 to 85 years. The original version created by these authors consists of 27 items with Likert-type responses ranging from 1 (not at all true) to 10 (totally true); since then, there have been several versions in which the number of items has been adapted or the response range has been reduced (Kalhori et al., 2015; Montag et al., 2015; Sar & Isiklar, 2012). The original total score ranged from 27 to 270 points and consisted of five factors: ‘Tolerance’, ‘Escape from everyday problems’, ‘Abstinence’, ‘Craving’ and ‘Adverse or negative consequences’. In our case, in 2014, we utilized the MPPUS as adapted by López-Fernández et al. (2012) for use among students and adolescents; the authors reduced the total items to 26 and modified the wording of other items. For the 2014 study (De-Sola et al., 2017a), we adapted the scale to the whole general population in Spain; the minimum score was 26, and the maximum score was 260, with higher scores indicating an increase in problematic use. The mean obtained was 68.9 (SD = 36.89), with a range of 234. Likewise, we obtained four factors that explained 59.8% of the total variance (‘Abuse and excessive use of a mobile phone’, ‘Loss of control’, ‘Craving induced by social context’ and ‘Tolerance’), of which only the first three really were useful; the fourth contained only one item (‘I never have enough time for my cell phone’). The internal consistency was also very adequate (α = 0.939), reflecting stability of the instrument (De-Sola et al., 2017a). Regarding the categorization of the users and following the criteria of previous studies, we established four categories (casual, regular, at-risk and problematic) considering as cutoff points the 15th, 80th and 95th percentiles, corresponding with scores of 33, 97 and 139 on the scale.

Based on these findings, we decided to revise the procedure. A factorial analysis using the 2014 dataset was performed to evaluate item performance, following recommended criteria for communalities and factor loadings (Costello & Osborne, 2005). Item 1 (“I never have enough time for my mobile phone”) showed no salient loading on any of the three extracted factors and exhibited a very low communality (h^2^ = 0.185), indicating that only 18.5% of its variance was shared with the common factors. Item 16 (“If I didn’t have a mobile phone, my friends would find it difficult to contact me”) loaded on Factor 3 (λ = 0.637) but presented the lowest communality in that factor (h^2^ = 0.439), lying at the lower bound of acceptable values. Conceptually, both items were also misaligned with the construct of problematic or addictive use: Item 1 reflected aspirational desire rather than symptomatology, and Item 16 captured a generalized technological norm rather than dysfunctional behavior. For these statistical and conceptual reasons, both items were excluded from subsequent analyses.Thus, we proceeded to eliminate items 1 and 16 in the 2018 and 2024 surveys, given their limited contribution to the factorial structure. The scale, therefore, was reduced to 24 items, ranging from 24 to 240 points, with a mean score of 88.65 points (SD = 49.96). With this, we achieved an increase in the reliability and stability of the instrument (α = 0.970).

Regarding the internal validity, after conducting a new factorial analysis using principal components and varimax rotation, we obtained three factors that explained 71.2% of the variance. These factors are practically the same as those in the previous investigation, with the exception of the disappearing of the fourth factor, which only contained one item. The final rotation converged in 10 iterations. All items had factor loadings greater than 0.50, with the Kaiser-Meyer-Olkin measure of sampling adequacy being 0.97 (KMO = 0.975), while Bartlett’s test of sphericity provided a chi square of 35,428.870 (gl = 276; *p* < 0.000), which confirmed the sampling adequacy as well as the suitability of the analysis. Thus, the first factor, *‘Mobile phone abuse and excessive use’*, which explains 29.5% of the variance, includes items 3, 5, 13, 14, 15, 16, 18, 19, 20, 21, 22 and 23 and refers to excessive use, recurring thoughts, changes in mood when a device is not available, social problems and interference in daily life, discomfort, awareness of abuse and warnings from people in one’s environment. The second factor, ‘*Loss of control*’, which explains 26.3% of the variance, includes items 1, 2, 4, 7, 8, 9, 10 and 17 and considers the problems derived from the progressive abandonment of activities, the inability to self-control, and the use of a cell phone to compensate for dysphoric moods. Finally, the third factor, *‘Craving induced by social context’*, which explains 15.4% of the variance, includes items 6, 11, 12 and 24 and reflects the perception of cell phone dependence in relevant social environments. The same categories for users (casual, regular, at-risk and problematic users) and the same cutoff criteria (15th, 80th and 95th percentiles, corresponding to scores of 35, 133 and 183) were used. However, to facilitate a global analysis, we added a new category, ‘users with difficulties’, which is the sum of at-risk and problematic users. Based in this analysis, the corrected scale was also set for the survey of 2024.

A detailed analysis of each item, indicated a higher score for items 1 (M = 5.08, SD = 2.82) (‘Sometimes, when I have felt bad, I have used my cell phone to feel better’) and 9 (M = 4.98, SD = 2.86) (‘I have used a cell phone to talk to others when I felt lonely or isolated’), which reflects the relevance of cell phones as a resource for coping with dysphoric moods. Similarly and considering users with difficulties (n = 316), i.e., the sum of at risk (n = 239) and problematic (n = 77) users, the items with higher scores were 8 (M = 7, 73, SD = 4.80) (‘I notice that the time I spend with my mobile phone has increased more and more’), 1 (M = 7.67, SD = 5.08) (‘Sometimes, when I have felt bad, I have used my cell phone to feel better’), 9 (M = 7.67, SD = 4.98) (‘I have used my cell phone to contact others when I felt lonely or isolated’) and 7 (M = 7.64, SD = 4.81) (‘Sometimes, when I am on my cell phone, I get carried away and do not pay attention to what I am doing at the time’). These results indicate a progressive lack of control with mobile devices as well as their use in confronting dysphoric feelings and social isolation (Table 2).

Regarding the MPACS scale, we conducted a new review to investigate the consistency, reliability and validity of the scale using the data of 2018 survey. The scale consists of eight items with Likert-type responses, ranging from 1 to 10 points, depending on the conformity and degree of situational concern, with eight statements referring to a hypothetical present moment when a cell phone cannot be used. The overall score ranges from 8 to 80, with a mean of 22.85 (SD = 14.85), with higher scores indicating higher cravings. The MPACS also presented, from the beginning, good internal consistency (α = 0.919) as well as factorial unidimensionality, i.e., a single factor that explained 64.3% of the variance, which in this case indicated that craving was a unique construct. Likewise, the scale exhibited construct validity through significant correlations with the total MPPUS (r = 0.785) and with each of the factors (r = 0.412 with factor I ‘Mobile phone abuse and excessive use’; r = 0.440 with factor II ‘Loss of control’; and r = 0.542 with factor III ‘Craving induced by social context’). Thus we keep the use of the original scale for the comparative surveys of 2014, 2018 and 2024.

### 3.2. Prevalence and Evolution of Problematic Mobile Phone Use

The analysis of the evolution of problem use indicated that there have been clear changes in the last decade on the prevalence of the different categories of users obtained using the MPPUS, when comparing the prevalences of the three surveys (Chi-square (df, 6) = 33.6, *p* < 0.001, Table 3). The main changes observed were the reduction in casual users and the increase in the number of regular users in 2024 with respect to 2014, the progressive reduction in problematic users in 2024 and the stabilization of the number of at-risk users along the decade (Chi-square (df, 3) = 17.8, *p* < 0.001, Table 3). Taking into consideration the category of users with difficulties, the prevalence change from 20.5% in 2014, to 19.6 in 2018, being significatively reduced in 2024 to 18.8%.

Considering the mean MPPUS scores, two-way ANOVA reflected a clear interaction in severity categories × year indicating that both, at risk users and problematic users had significative lower scores in 2024 than in 2018 (*F*(4,4292) = 5.69, *p* < 0.001). The reduction in the prevalence of problematic mobile phone use is reflected in the score of the users with difficulties (combined category including at risk and problematic users). that decreased from 167.99 in 2018 to 161.37 in 2024.

#### 3.2.1. Differences by Age and Gender

Table 4 reflects the prevalence of each category of the MPPUS regarding age interval. Table 5 shows these differences but in the mean MPPUS score in each age group. Finally, Table 6 shows gender differences in MPPUS scores regarding age interval.

Results clearly show that MPPUS prevalence of both problematic use and users with difficulties is higher in young people ranging from 16 to 35 years old. Data shows the different proportions of each MPPUS categories, where we can see how most users are regular ones, and that the prevalence of users with difficulties decreases with age, being the highest prevalence found at groups from 16 to 35 years of age. One way ANOVA of mean MPPUS scores supported these findings both in 2018 (*F*(9,1602) = 32.6, *p* < 0.001) and 2024 (*F*(9,1995) = 43.5, *p* < 0.001), Table 4 and Table 5.

Gender analysis revealed the same pattern in the distribution of mean MPPUS scores. Interestingly, there was a small change in mean MPPUS scores when comparing 2018 with 2024. While in both the 2014 and 2018 surveys there were no major gender differences (only significantly higher scores in males aged 26–45 years), in 2024 these differences disappeared, and a significant increase in mean scores emerged in the 16–20-year age group, which was higher among females (Table 6). The only consistent finding in both the 2018 and 2024 surveys was the highest MPPUS scores observed among women in the oldest age group, 61–65 years, indicating a more active use of mobile phone technology in older women.

#### 3.2.2. Differences by Education Level and by Main Occupation

Although mean scores varied slightly between educational groups, these differences did not reach statistical significance across education levels in either 2018 or 2024 (Table 7). Prevalence analysis indicated that the increased prevalence of users with difficulties observed among those with primary/basic education in the 2018 survey disappeared in 2024 (Supplementary Table S1). These results indicate that mobile phone use has permeated all levels of society, regardless of the level of education attained.

Considering profession or main activity (retired—without work or without specific pursuits, domestic work, studying or working), only students show a significantly higher mean MPPUS scores (Table 8) and prevalence (Supplementary Table S2) as users with difficulties when compared with the total. Two-way ANOVA revealed differences in MPPUS scores regarding the level of occupation (*F*(3,3609) = 54.3, *p* < 0.001), as well as small differences regarding the year of study (*F*(1,3609) = 4.1, *p* < 0.05). Prevalence of users with difficulties stands around 28% of the total students, both in 2018 and 2014. These differences stem from at-risk use, not problematic use. In contrast, those users without a specific pursuit report more casual use of mobile phones, with very little problematic use (Supplementary Table S2).

#### 3.2.3. Differences by Habitat

In the analysis by population center (rural areas vs. urban areas), urban areas were considered those with a population size equal to or greater than 50,000 inhabitants. In contrast, rural areas comprise a population below this size. While in 2018, prevalence of users with difficulties, especially at-risk users, is significantly higher in urban areas than rural areas (Table 9), these differences disappeared in 2024, again suggesting a complete permeation of mobile phone technology in all habitats.

### 3.3. Evolution of Craving for Mobile Phone Use (MPACS)

Regarding craving as measured by the MPACS, we observed that the total mean score in this study, both in 2018 and 2024, was significantly higher than that previously reported (De-Sola et al., 2017b). In other words, cravings associated with problematic cell phone use have increased over time. The analysis by user type showed that this increase was significant across all categories except among casual users (Table 10). For the mean MPACS score, the increase relative to 2014 was significant for both 2018 and 2024 (*F*(2,4336) = 265.8, *p* < 0.001). Among problematic users, the ANOVA revealed a more pronounced pattern of growth (*F*(2,182) = 16.14, *p* < 0.001), with all three survey periods differing significantly from each other, reflecting a time-related increase in the severity of craving (Table 10).

### 3.4. Factors Influencing Problematic Mobile Phone Use

In the study of 2014, we identified certain variables contributing to problematic mobile phone use (age and hours of use), as well as several disorders associated with more severe scores of problematic mobile phone use including anxiety state and alcohol consumption, but not depression. We have included them in the 2024 study, together with an analysis of internet gaming disorder, by using the Igsd9 scale in its short form of 9 items. The results of the correlations of the MMPUS scores with these variables is depicted in Table 11. All variables, except for depression scores, correlate significantly with the MPPUS score. MPPUS scores correlate positively with the number of hours spent on the mobiles phone, anxiety scores and with the AUDIT-C scores for alcohol consumption. Age presented a negative correlation indicating that younger ages had greatest scores for MPPUS. No correlation with depression was observed. These results are similar with those of 2014, indicating that these are solid factors contributing to problematic mobile phone use. In the case of videogaming, only 440 participants declared using the mobile phone for gaming. In this population, the association of MPPUS with the score of Igsd9-sf was strong, as revealed by the correlation analysis (Table 11).

**Figure 1:**
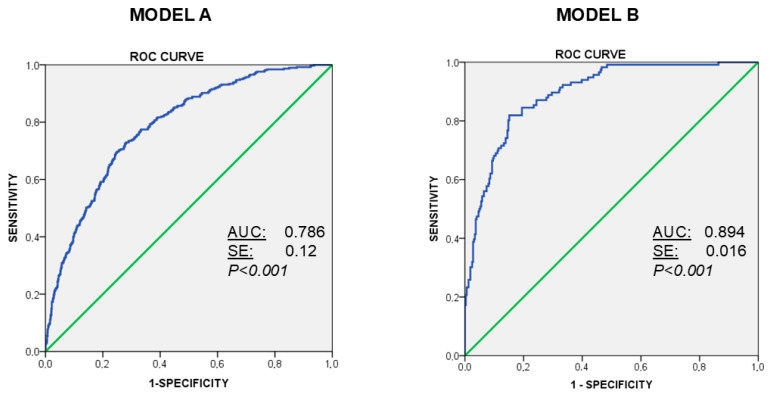
ROC curves for multivariate predictive models of problematic mobile phone use (category users with difficulties). **Model A**, performed with data of all participants, includes in the final analysis the variables Age, Number of hours of use of the mobile phone, STAI-S anxiety score and AUDIT-C scores for alcohol consumption. **Model B**, performer from data of videogamers, includes Age, Sex (male category), Number of hours of use of the mobile phone, STAI-S anxiety score and internet gaming disorder score (Igds9-sf).

Based on the 2014 study and using the data from the survey of 2024, we generated a binary logistic regression model to discriminate between users with or without difficulties to control the use of the mobile phone (assessed by MPPUS) The analysis can be found in Table 12. In the model A (Whole participants), the variables included in the first step were “age”, “Gender”, “Hours of Use”, “Habitat”, “Educational Level”, “Anxiety Score (STAI)”, “Depression Score (BDI)”and “Alcohol Consumption (AUDIT-C score)”. In the model B (videogamers, n = 440), the variables included in the first step were “age”, “Gender”, “Hours of Use”, “Habitat”, “Educational Level”, “Anxiety Score (STAI)”, “Depression Score (BDI)”, “Alcohol Consumption (AUDIT-C score)” and “Videogaming Score (Igds9-sf)”. The models were prepared using the backward stepwise method and the predictive covariates were (a) restricted to four in the general model A (“age”, “Hours of Use”, “Anxiety Score”, “Depression Score”and “Alcohol Consumption”), and to (b) five in Model B-videogamer population (“age”, “Gender”, “Hours of Use”, “Anxiety Score”, and “Videogaming Score”. In Model A, The Hosmer–Lemeshow test indicated good calibration (X^2^ = 3.98; df = 8; *p* = 0.858) it had a sensitivity of 0.73 and specificity of 0.45. ROC curve analysis (AUC = 0.796) indicated an acceptable discrimination power (Figure 1). In Model B, The Hosmer–Lemeshow test indicated good calibration (X^2^ = 5.905; df = 8; *p* = 0.658) it had a sensitivity of 0.82 and specificity of 0.67. ROC curve analysis (AUC = 0.894) indicated a high discrimination power (Figure 1).

In Model A, which included all participants, younger age, more hours of daily phone use, higher anxiety scores, and higher alcohol consumption were associated with increased odds of belonging to the ‘users with difficulties’ group. In contrast, Model B, restricted to videogamers, highlighted a strong incremental effect of gaming severity: higher IGDS9-SF scores almost doubled the odds of problematic phone use even after controlling for age, sex, hours of use, and anxiety. These results underline the combined contribution of general overuse, emotional distress, and problematic gaming to problematic mobile phone use.

## 4. Discussion

The main objective of this research was to analyze the evolution of problematic mobile phone use in the last decade in Spain by comparing the results of our previous survey done in 2014 (De-Sola et al., 2017a, 2017c) with new data collected in 2018 and 2024 in a representative sample of the Spanish population, i.e., individuals who are 16 to 80 years of age. In our previous study, we used the MPPUS (De-Sola et al., 2017b) adapted for Spain (López-Fernández et al., 2012). We obtained four factors (‘Cell phone abuse and excessive use’, ‘Loss of control’, ‘Craving induced by social context’ and ‘Tolerance’), of which only the first three were useful; the fourth only contained one item. Likewise, the internal consistency was very adequate, showing stability of the instrument. Under the hypothesis that we were facing behavior that could be comparable to addiction, we also developed the MPACS (De-Sola et al., 2017b) to verify the existence of cravings related to dependence derived from mobile phone abuse. This scale, based in part on the work by López Durán and Becoña Iglesias (2006) with subjects dependent on cocaine, aims to assess the existence of cravings related to the problematic use of mobile phones through assessments of concern and anxiety via eight hypothetical situations in which the device cannot be used. The MPACS, since beginning developed, has exhibited good internal consistency as well as factorial unidimensionality, with craving as a single construct; construct validity was exhibited through significant correlations with the MPPUS.

In the present study, with a broader representative sample and based on the previous analyses, we eliminated two items (1 and 16) from the MPPUS, given their scarce contribution to the previous factorial structure, and we revised and adapted the wording of some items after the original pilot study. The scale was reduced to 24 items, thereby increasing reliability and stability. A new factorial analysis offered us a more refined final structure, with greater explained variance and three factors: ‘Mobile phone abuse and excessive use’, which refers to excessive use, recurring thoughts, alterations in mood when a cell phone is not available, social problems and interference in daily life, discomfort, awareness of abuse and warnings from people in one’s environment; ‘Loss of control’, which refers to the problems derived from the progressive abandonment of activities, the inability to self-control, and the use of a cell phone as a resource to compensate for dysphoric moods; and ‘Craving induced by social context’, which refers to mobile phone dependence in social environments.

The item analysis of the MPPUS, taking into account the highest scores, indicates three axes as the basis for cell phone abuse: perception of poor self-control capacity with devices (Goswami & Singh, 2016; Jones, 2014), cell phone use as a means of coping with dysphoric moods (Demirci et al., 2015) and cell phone use as a means of relieving feelings of social isolation of not losing contact with one’s environment. These axes are consistent with factors such as dependence on the social environment and the need for belonging (Walsh et al., 2011), for which cell phones are seen as vital vehicles of contact (Shambare et al., 2012; Takao et al., 2009; Walsh et al., 2010). Additionally, we used the same user categories (casual, regular, at-risk and problematic users) adding in this case and in order to provide a better overall appreciation of the prevalence, the category ‘users with difficulties’, i.e., the sum of at-risk and problematic users.

Regarding the MPACS in the current study and after repeating the previous analyses without having made any modification to the scale, the results showed an improvement in internal consistency, and the unidimensional factorial structure was confirmed, with greater explained variance. Furthermore, Ozer and Özer (2018) subsequently confirmed the unidimensionality, stability and internal consistency of the MPACS in a population sample of individuals 18 to 65 years of age in Turkey. A detailed analysis of the highest scoring items indicates that cravings related to devices are associated both with situations in which the terminal may not be available as well as in situations of prohibition or the impossibility of using a device at certain times. The MPACS again showed adequate construct validity, with significant correlations with the total MPPUS score as well as with its three factors separately; the correlation with ‘Craving induced by social context’ demonstrates the importance of the context in problematic cell phone use, similar to the importance of context in addictions in general (Griffiths, 2005; Tiffany, 1990; Weaver & Schnoll, 1999; West, 2011).

Using these two scales, the revised MPPUS and the MPACS, we conducted new analyses in 2018 and 2024, compared to the previous study, to examine the prevalence of problematic mobile phone use and the cravings associated with it in Spain. Overall, considering the four categories of users, the results indicate that the prevalence of both at-risk users and problematic users has decreased over the past decade. However, although this decrease is moderate, 18.8% of the population still experiences difficulties in controlling their mobile phone use, indicating that abuse and problematic use remain a stable reality in Spanish society. Moreover, this reduction in prevalence is accompanied by a consistent increase in MPACS scores, suggesting that the subjective loss of control over mobile phone use is associated with greater craving for its use, reflecting an increased severity of mobile phone addiction. At first glance, the simultaneous decrease in the prevalence and mean MPPUS scores among problematic users and the increase in MPACS scores may appear contradictory. One possible explanation is that, over time, problematic mobile phone use has become more concentrated among a smaller group of users who experience more intense craving and loss of control, while less severe cases may have normalized or adapted their usage patterns. In other words, the overall prevalence of “users with difficulties” has slightly declined, but those who remain in this category report higher craving and dependence, consistent with a more severe clinical profile. Additionally, the higher “craving” scores observed in both regular and problematic users may reflect the growing integration of mobile phone use into everyday life, with this tendency becoming more pronounced among problematic users. In line with recent epidemiological evidence, it is also important to avoid over-pathologizing normative patterns of technology use. Large-scale studies comparing DSM-5-inspired versus ICD-11-inspired approaches have shown that liberal cut-off scores can markedly inflate prevalence estimates of problematic Internet use and gaming, whereas more conservative criteria yield more realistic rates and identify a smaller subgroup with clear functional (Nogueira-Lopez et al., 2023). Our pattern of lower prevalence but higher craving among those classified as “users with difficulties” is consistent with this recommendation, suggesting that this label is increasingly restricted to individuals presenting a more clearly defined clinical profile rather than milder or transient patterns of heavy use.

Considering age, individuals between 16 and 35 years exhibit the highest prevalence of problematic mobile phone use throughout the decade. This prevalence progressively decreases after the age of 35, giving way to more casual use. However, it is important to note that the number of regular users has increased across all adult age groups over 35, suggesting that mobile phone use has permeated all levels of society and poses a potential risk of future transition toward problematic use among the adult population. This concern has already been highlighted by Smetaniuk (2014) and may be relevant for designing interventions that extend beyond the traditional focus on young people and adolescents. The growing range of mobile applications integrated into daily life—such as social networks, video games, instant messaging services like WhatsApp, and photo- and selfie-sharing platforms—all with significant addictive potential, has indeed contributed to this increase (Cha & Seo, 2018; Fransson et al., 2018; Kuss & Griffiths, 2017; Starcevic et al., 2018).

Regarding gender, as in our previous study, there are no significant overall differences between men and women. This does not mean that such differences do not exist but rather that they are likely related to age group, type of device, or specific needs. In this regard, a detailed analysis of MPPUS scores showed that the small but significant difference previously observed in 2018—where male users aged 26 to 45 scored higher—had disappeared by 2024. The only difference that persisted across both studies was that female users over 60 years of age consistently showed higher MPPUS scores than males. In the latest survey, younger female users (16–20 years old) also scored higher on the MPPUS than their male counterparts. Regarding other factors, Foerster, Roser, Schoeni, and Röösli (Foerster et al., 2015), among others, have suggested various patterns of mobile phone use, including dependence on social contact, which appears to be more strongly associated with women. In general, social anxiety (Pierce, 2009; Sultan, 2014) and dependence on one’s social environment (Igarashi et al., 2008; Walsh et al., 2011; Walsh et al., 2010) are closely linked to mobile phone dependence, occurring more frequently among women due to their greater engagement in interpersonal relationships through interactive social applications (Valkenburg & Peter, 2007). In our own data, we also observed these gender differences, particularly in relation to the types of devices and applications used (De-Sola et al., 2019). Further analysis of this sex-dependent pattern is needed to fully understand the contribution of gender to problematic mobile phone use.

With respect to education level, no overall differences were observed. Mobile phone use has become widespread in society, largely independent of the degree of education attained. In this regard, a lack of education or a low educational level shows some association with mobile phone overuse but does not appear to be a decisive factor in problematic use. However, research in this area has yielded mixed results. Sahin et al. (2013), López-Fernández et al. (2012), and Leung (2007), among others, found a relationship between lower socioeconomic and educational levels and problematic mobile phone use. Nevertheless, these differences seem to be more closely related to age, since analysis by main occupation revealed that only students show a significantly higher prevalence of users with difficulties—stemming from at-risk use rather than problematic use—following the same pattern in both the 2018 and 2024 surveys (see Supplementary Materials). In contrast, individuals without specific occupations, as well as those engaged in domestic tasks, reported more casual or regular mobile phone use and very little problematic use. These findings, consistent with our previous studies (De-Sola et al., 2019, 2017a, 2017b), suggest that although employment may increase mobile phone use, it is not, in itself, a factor that promotes problematic use. Regarding population size, the prevalence of “users with difficulties,” particularly those at risk, was higher in urban areas in 2018; however, this difference disappeared by 2024, suggesting a broad diffusion of mobile phone use across both urban and rural areas, although specific usage patterns may still vary between settings.

Finally, to further re-evaluate the main factors contributing to problematic mobile phone use, we examined variables identified in previous studies (De-Sola et al., 2019, 2017c), including age, the number of hours of phone use per day, the presence of anxiety or depression, and alcohol consumption. In addition, given the close association between mobile technology and Internet Gaming Disorder (Li et al., 2026; Menéndez-García et al., 2022), we assessed the presence of abnormal scores related to video gaming. The data indicate that younger age, a high number of hours of phone use, and the presence of anxiety (but not depression) are major determinants of problematic mobile phone use, with alcohol consumption playing a minor role. These variables also showed predictive value, as revealed by binary logistic regression (Model A) and ROC curve analysis (see Figure 1). These findings are consistent with those of the 2014 study, particularly regarding the relevance of state anxiety, which has also been identified as a major contributing factor in studies conducted in other countries using different instruments (Li et al., 2026; Lu et al., 2025).

These findings are consistent with theoretical accounts emphasizing self-regulation deficits and maladaptive coping in behavioral addictions (Billieux et al., 2015; Brand et al., 2019). According to the I-PACE model (Brand et al., 2019), individuals with heightened negative affect and poor executive control may increasingly rely on specific online activities or digital devices as a strategy to regulate dysphoric moods and to maintain a sense of social belonging. Longitudinal evidence in children and adolescents further supports this framework, showing that problematic Internet use and emotional variables such as depression, anxiety, and emotional symptoms are dynamically related over time, with bidirectional influences between emotional distress and problematic use (Villanueva-Blasco et al., 2026). Within this perspective, using the mobile phone as a means of mood regulation and social compensation may act as a maladaptive coping strategy that progressively strengthens addictive patterns, which is reflected in the craving component captured by the MPACS (De-Sola et al., 2017b).

When focusing on the subgroup of participants who reported playing video games (n = 440), we found a clear association between IGDS9-SF scores and MPPUS scores, reflected in nearly double the scores compared with regular users (Table 11). Binary logistic regression in this subgroup (Model B; Table 12 and Figure 1) revealed a strong influence of video gaming as a major contributing factor to problematic mobile phone use. Similar findings have been reported in other studies (Marshall et al., 2024; Menéndez-García et al., 2022; Zhang et al., 2024), which suggest the need for parental involvement and preventive interventions to address both issues. The present study shows us that problematic cell phone use is a phenomenon that has definitively been installed in our lives, probably due to a social environment that increasingly offers more comfort but also more dependence and technological demand. Although this phenomenon initially affected youth, today it also affects the adult population. These data point to worrying trends, given that some studies have demonstrated (Marshall et al., 2024; Zhou et al., 2014) the power of parents against the abuse of technologies among young people. Currently, although the population prevalence is slightly decreasing, the data also indicate that dependence and cravings are increasing, leading us to propose that mobile phones have not only become commonplace in our lives but have also become unstoppable means for new styles of interaction and communication. It is evident therefore that mobile phones are no longer just devices but, currently, instruments without which we cannot live and whose unwanted side effects need to be understood and prevented.

### Strengths and Limitations of This Study

More research is needed that establishes causal relationships, that directly and objectively consider the mediating variables and that analyze in greater depth the specific uses that determine problematic cell phone use. Future longitudinal panel studies following the same individuals over time would be particularly valuable to establish causal links between changes in psychological variables (e.g., anxiety, gaming) and trajectories of problematic mobile phone use. The data for this research was obtained through an online questionnaire using the most efficient self-registration systems, free of subjectivity in assessments (Boase & Ling, 2013; Kwon et al., 2013; Lin et al., 2015; Montag et al., 2015; Roberts et al., 2014; Shin & Dey, 2013; Tosell et al., 2015). To this bias and limitation, we must add the self-awareness of abuse that mobile users usually have, which adds more subjectivity to our results. On the other hand, the greatest strength of this research is its sample size and representativeness of the Spanish population, covering practically the entire national territory, two serial surveys separated by 5 years, and the comparability with the previous study in 2014 using the same design, allowing to characterize the evolution of the problem cell phone use in the last 10 years.

A further limitation is that, although we examined predictors separately in age- and gender-stratified analyses, we did not specify formal interaction terms (e.g., gender × age, gender × hours of use, gender × gaming severity) in our multivariable models. The primary aim of the present study was to identify robust overall associations between sociodemographic and psychological variables and problematic mobile phone use that could be compared across the 2018 and 2024 surveys, rather than developing a fully saturated interaction model. Given the number of candidate predictors and outcomes, adding multiple interaction terms would have substantially increased model complexity, the risk of overfitting, and the burden of multiple testing, potentially hindering the interpretability and comparability of the results. Future research specifically designed and powered to address moderation questions should explicitly model these interaction effects to clarify how demographic and clinical variables shape vulnerability to problematic mobile phone use.

## 5. Conclusions

The regular use of mobile phone technology has become firmly established in Spain, as in most modern societies, and is largely independent of sex, education level, occupational status, or place of residence. Problematic mobile phone use remains a prevalent issue, although its overall prevalence has slightly decreased over the past decade. However, problematic users show greater severity in their loss of control over mobile phone use, as reflected by a persistent increase in craving scores. Major factors contributing to problematic phone use include younger age, the number of hours of use, engagement in video gaming, and the presence of negative mood states such as anxiety. The present study underscores the need to implement effective interventions to prevent the unwanted consequences of mobile phone technologies.

## Figures and Tables

**Table 1:** Sociodemographic data for the sample (2018 and 2024).

Regional Distribution	2018	2024	Population by Inhabitants	2018	2024	Age	2018	2024
Andalusia	17.9%	18%	Up to 2000	3.2%	2.3%	16 to 20 years	8.1%	10.4%
Aragon	2.9%	2.8%	From 2001 to 5000.	4.7%	3.0%	21 to 25 years	12.0%	29.6%
Asturias	2.2%	2.2%	From 5001 to 10,000	5.3%	5.2%	26 to 30 years	11.9%	10.5%
Balearic Islands	2.3%	1.8%	From 10,001 to 50,000	17.1%	20%	31 to 35 years	11.1%	18.9%
Canary Islands	4.5%	4.9%	From 50,001 to 200,000	15.7%	19.3%	36 to 40 years	11.0%	3.1%
Cantabria	1.2%	1.2%	From 200,001 to 500,000.	6.1%	6.4%	41 to 45 years	10.0%	5.0%
Castilla La Mancha	4.4%	4.0%	More than 500,000	47.9%	43.7%	46 to 50 years	10.1%	5.9%
Castilla León	5.1%	5.2%				51 to 55 years	9.0%	6.3%
Catalonia	16.2%	16.5%	**Population size**			56 to 60 years	8.9%	4.8%
Extremadura	2.3%	2.0%	Rural areas	30.2%	30.6%	61 to 65 years	7.9%	5.5%
Galicia	5.8%	5.8%	Urban areas	69.8%	69.4%			
La Rioja	0.7%	0.6%	**Gender**			**Mean age (years) **	39.6	33.9
Madrid	14.6%	14.6%	Women	50.1%	50%	**SD age years**	13.6	13.6
Murcia	3.1%	3.1%	Men	49.9%	50%	**Main activity**		
Navarre	1.5%	1.4%				Working	62.5%	62.8%
Basque Country	4.7%	4.7%	**Studies**			Studying	13.3%	22.6%
Valencian Community	10.6%	11.0%	Higher, university	51.7%	52.2%	Housework	7.6%	2.7%
			Middle, high school	39.9%	40%	No activity,	16.6%	11.9%
			Basic or uneducated	8.4%	7.8%			

**Table 2:** Factorial analysis of the MPPUS.

	**1**	**2**	**3**
13. I usually dream of my cell phone.	0.802		
16. I feel discomfort associated with cell phone use.	0.773		
19. More than once, I have been late somewhere because I am hooked to my cell phone.	0.735		
14. My friends and family complain because I use my cell phone a lot.	0.732		
15. My performance has decreased as a result of the time I spend with my cell phone.	0.722		
20. I get in a bad mood when I am forced to turn off or disconnect my phone somewhere.	0.719		
21. I have been told that I spend too much time on my cell phone.	0.684		
18. Sometimes, I would rather be on the phone than deal with other more urgent issues.	0.671		
5. I have sometimes incurred significant financial expenses because of my cell phone.	0.613		
22. More than once, I have been in a pinch because my cell phone has started to ring in a place or at a time when I should have it turned off or disconnected.	0.595		
3. More than once, I have lied or hidden from others the time I am on the phone.	0.571		
23. My friends do not like me to have my phone turned off or disconnected.	0.530		
8. I notice that the time I spend with my cell phone has increased more and more.		0.795	
2. I spend a lot of time on my cell phone when I should be doing other things, and that causes problems for me.		0.784	
1. Sometimes, when I have felt bad, I have used my cell phone to feel better.		0.747	
7. Sometimes, when I am on my cell phone, I get carried away and do not pay attention to what I am doing at the time		0.745	
4. My cell phone use has deprived me of sleep.		0.696	
9. I have used my cell phone to contact others when I felt alone or isolated.		0.678	
17. I see myself hooked to my cell phone longer than I would like.		0.651	
10. I have tried to spend less time with my cell phone, but I am unable.		0.562	
6. When I do not have a mobile phone, I worry about missing a call or message.			0.769
24. I feel lost without my cell phone.			0.664
11. I find it difficult to turn off, mute or disconnect my cell phone.			0.664
12. I feel nervous if I spend time without checking my messages or if I have not connected my cell phone.			0.624
	**1**	**2**	**3**
Eigenvalue of the factors or components rotated	7.089	6.320	3.689
Percentage of variance explained by each component or factor	29.538	26.332	15.373
Cumulative percentage of variance explained by the components or factors	29.538	55.869	71.242

**Table 3:** Prevalence and mean scores by type of user, based on the MPPUS.

	Mean MPPUS Score	SD	Maximum Score	Minimum Score	Range	Prevalence
**Casual users**						13.6% (2014) ^1^
**2018**	28.91	3.78	35	24	11	**15.1%**
**2024**	29.04	3.53	35	24	11	**9.6%**
**Regular users**						65.9% (2014) ^1^
**2018**	78.62	28.69	133	36	97	**65.3%**
**2024**	80.88	27.10	133	36	97	**71.5%**
**At-risk users**						15.4% (2014) ^1^
**2018**	156.61	13.70	183	134	49	**14.8%**
**2024**	153.90	14.10	183	134	49	**15.6%**
**Problematic users**						5.1% (2014) ^1^
**2018**	203.31	15.32	240	184	56	**4.8%**
**2024**	197.23	12.83	240	184	56	3.2% (*)
**Users with Difficulties**						20.5% (2014) ^1^
**2018**	167.99	24.53	240	134	106	**19.6%**
**2024**	161.37 **(#)**	21.47	240	134	106	18.8% (*)
**Global Mean Score**						
**2018**	88.65	49.96	240	24	216	
**2024**	91.05	44.61	240	24	216	

^1^ Data from Reference (De-Sola et al., 2017b). (*) *p* < 0.01 2024 versus 2014. (#) *p* < 0.01 2024 versus 2018.

**Table 4:** Prevalence of MPPUS categories by age interval.

	Age Interval										
	16–20	21–25	26–30	31–35	36–40	41–45	46–50	51–55	56–60	61–65	Total
**Casual users**											
**2018**	2.3% **	2.1% **	5.2% **	4.5% **	11.9%	14.3%	20.2%	26.9% *	34.7% **	40.6% **	15.1%
**2024**	2.6% **	2.4% **	7.6% *	6.1% *	11.3%	14.0% *	19.3% **	22.0% **	26.0% **	34.2% **	9.6%
**Regular users**											
**2018**	69.5%	70.5%	63.9%	67.0%	66.7%	67.1%	64.4%	62.8%	61.8%	57.0%	65.3%
**2024**	67.8%	69.7%	68.6%	80.4% *	69.4%	74.0%	73.1%	70.9%	68.8%	64.0%	71.5%
**At-risk users**											
**2018**	24.4% *	21.2% *	26.2% **	18.4%	16.4%	12.4%	11.0%	6.9% **	2.1% **	2.3% **	14.8%
**2024**	23.1% *	22.9% *	20.0%	11.96%	17.7%	11.0%	5.9% **	5.5% **	5.2% **	0.9% **	15.6%
**Problematic users**											
**2018**	3.8%	6.2%	4.7%	10.1% *	5.1%	6.2%	4.3%	3.4%	1.4% *	0.0% **	4.8%
**2024**	6.7% *	5.1%	3.8%	1.6%	1.6%	1.0% *	1.7%	1.6%	0.0% **	0.9% *	3.2%
**Users with difficulties**											
**2018**	28.2% **	27.5% **	30.9% *	28.5% *	21.5%	18.6%	15.3%	10.3% **	3.5% **	2.3% **	19.6%
**2024**	29.8% **	28.0% **	23.8% *	13.6%	19.3%	12.0%	7.6% **	7.1% **	5.2% **	1.8% **	18.8%
**Total**											
**2018**	131	193	191	179	177	161	163	145	144	128	1612
**2024**	208	590	210	378	62	100	119	127	96	111	2001

(*) Significant percentage difference (*p* ≥ 0.05) with respect to the total/(**) Significant percentage difference (*p* ≤ 0.01) with respect to the total.

**Table 5:** Mean scores of MPPUS by age interval.

	16–20	21–25	26–30	31–35	36–40	41–45	46–50	51–55	56–60	61–65	Total
**Mean (M)**											
**2018**	105.93 **	105.60 **	107.46 **	109.77 **	92.51	87.74	80.35 *	67.48 **	58.05 **	52.56 **	**88.65**
**2024**	113.24 **	107.63 **	98.17	88.13	85.06	78.81 **	68.83 **	68.24 **	62.20 **	52.12 **	91.36 **
**Standard deviation (SD)**											
**2018**	42.48	46.07	48.87	51.31	50.46	51.06	49.07	44.10	36.13	29.67	**49.96**
**2024**	41.51	44.36	44.33	39.73	46.26	41.74	38.73	38.61	36.17	28.77	**45.13**
**Total**											
**2018**	131	193	191	179	177	161	163	145	144	128	**1612**
**2024**	208	594	210	378	62	100	119	127	96	111	**2001**

(*) Significant difference (Student’s t) (*p* ≤ 0.05) with respect to the total/(**) Significant difference (Student’s t) (*p* ≤ 0.01) with respect to the total.

**Table 6:** Mean MPPUS scores displayed by age and gender distribution.

	Age Interval										
	16–20	21–25	26–30	31–35	36–40	41–45	46–50	51–55	56–60	61–65	Total
**Mean 2018**											
**MALES**	101.79	101.92	115.51 *	121.35 **	93.04	95.13 *	80.13	65.30	55.19	46.42	**87.88**
**FEMALES**	109.12	107.78	101.40	98.31	91.99	78.60	80.63	70.39	61.23	61.53 **	**89.50**
**SD (n) 2018**											
**MALES**	36.49 (57)	43.71 (72)	50.91 (82)	50.35 (89)	50.31 (88)	50.01 (89)	48.81(92)	41.80 (83)	34.28 (76)	24.91 (76)	**50.31 (804)**
**FEMALES**	46.56 (74)	47.57 (121)	46.59 (109)	49.87 (90)	50.89 (89)	51.21 (72)	49.75 (71)	47.18 (62)	38.08 (68)	33.77 (52)	**49.61 (808)**
**Mean 2024**											
**MALES**	99.42	108.78	99.26	86.77	88.97	79.03	68.82	69.78	62.21	49.67	**82.75**
**FEMALES**	117.97 **	107.32	96.67	90.58	78.16	78.16	68.88	63.85	62.16	63.95 *	99.98 **
**SD (n) 2024**											
**MALES**	45.61 (53)	46.8 (125)	47.23 (121)	41.94 (243)	44.01 (36)	42.07 (75)	39.21 (87)	42.25(94)	36.54 (77)	25.12 (19)	**45.05 (1000)**
**FEMALES**	39.05 (155)	43.7 (469)	40.26 (89)	35.14 (135)	49.56 (26)	41.56 (25)	38.00 (32)	25.58 (33)	35.59 (19)	45.08 (92)	**43.55 (1001)**

(*) Significant difference (Student’s t) (*p* ≤ 0.05) males versus females within each age interval (**)/Significant difference (Student’s t) (*p* ≤ 0.01) males versus females within each age interval.

**Table 7:** MPPUS scores by education level.

	Without Education	Primary/Basic Education	Middle School, High School	Higher Education, University
**Casual Users**				
**2018**	26.5 (2.1)	29.3 (3.4)	27.7 (3.1)	28.0 (3.3)
**2024**	30 (0)	28.7 (3.8)	29.0 (3.5)	29.1 (3.6)
**Regular Users**				
**2018**	73.3 (9.2)	74.4 (29.3)	77.1 (29.9)	77.7 (28.9)
**2024**	88.3 (36)	80.8 (28.0)	81.4 (26.5)	80.6 (27.3)
**At-risk users**				
**2018**	151.7 (16.5)	157.0 (14.1)	154.9 (13.3)	157.8 (13.7)
**2024**	156.0 (13.6)	149.5 (15.7)	154.1 (13.9)	154.2 (14.0)
**Problematic Users**				
**2018**	198.5 (11.9)	198.6 (14.7)	206.3 (15.4)	206.3 (15.4)
**2024**	198 (0)	203.5 (15.7)	193.0 (8.2)	198.3 (14.1)
**Users with Difficulties **				
**2018**	**151.7 (16.5)**	**162.1 (18.9)**	**165.2 (23.0)**	**171.2 (26.1)**
**2024**	**164.4 (22.1)**	**164.9 (29.5)**	**160.9 (19.8)**	**161.1 (21.3)**
**Total users**				
**2018**	**9**	**126**	**644**	**833**
**2024**	**13**	**142**	**645**	**1201**

**Table 8:** Mean MPPUS scores displayed by main occupation.

	No Pursuits or Work	Housework	Studying	Working	Total
Mean (M)					
**2018**	66.14 **	82.39	106.62 **	91.53	88.65
**2024**	76.96 *	92.18	109.13 **	87.67	91.36
Standard deviation (SD)					
**2018**	39.79	48.91	42.02	51.92	49.96
**2024**	42.14	43.76	43.03	44.63	45.13
Total					
**2018**	**267**	**122**	**215**	**1008**	**1612**
**2024**	**238**	**55**	**453**	**1259**	**2001**

(*) Significant difference (Student t) (*p* ≤ 0.05) with respect to the total/(**) Significant difference (Student t) (*p* ≤ 0.01) with respect to the total.

**Table 9:** Prevalence and Scores of MPPUS Categories by Habitat (Rural *versus* Urban Areas).

		PREVALENCE		MEAN MPPUS		SD	
		Rural	Urban	Rural	Urban	Rural	Urban
**Casual ** **Users**	**2018**	15.6%	14.8%	29.07	28.83	3.79	3.78
**2024**		10.7%	9.2%	29.00	29.06	3.50	3.56
**Regular Users**	**2018**	69.4%	63.6%	78.76	78.55	29.72	28.40
**2024**		69.5%	72.4%	79.59	81.43	26.38	27.40
**At-risk users**	**2018**	11.7%	16.2% *	154.28	157.33	11.40	14.29
**2024**		16.4%	15.2%	153.45	134.11	13.75	14.29
**Problematic Users**	**2018**	3.3%	5.4%	202.25	203.59	18.83	15.04
**2024**		3.3%	3.2%	195.05	198.2	12.41	13.00
**Users with Difficulties**	**2018**	15.0%	21.6% **	164.79	168.94	23.65	24.75
**2024**		19.7%	18.4%	160.32	161.86	20.56	21.91
**Total **							
**2018**		N = 487	N = 1125	83.91	90.70 #	46.57	51.24
**2024**		N = 614	N = 1391	90.06	91.94	44.95	45.22

(*) Significant percentage difference (*p* ≤ 0.05)/(**) Significant percentage difference (*p* ≤ 0.01). (#) *p* < 0.01, Student’s *T* test versus rural areas.

**Table 10:** Mean comparative MPACS scores for 2014 and 2018.

	Previous Study—2014 ^(1)^ (n = 1126)		Current Study—2018 (n = 1612)		Current Study—2024 (n = 1601)	
	**Mean**	**SD**	**Mean**	**SD**	**Mean**	**SD**
**Casual users**	9.33	3.28	10.43	3.77	9.98	1.88
**Regular users**	19.98	11.42	21.96	12.02	24.88 **	6.61
**At-risk users**	37.82	10.83	38.58	13.14	44.94 **	5.20
**Problematic users**	50.57	14.67	57.41 **	10.59	62.74 **	6.46
**Users with Difficulties**	41.02	13.10	49.79 **	9.57	48.87 **	9.20
**Total**	22.85	14.85	27.17 **	16.79	34.22 **	16.01

^(1)^ Data from reference (De-Sola et al., 2017b). (**) Significant difference (Student t) (*p* ≤ 0.01) versus 2014 values.

**Table 11:** Correlation of MPPUS scores with main identified mobile phone problem use-contributing factor.

	Age	Hours of Use	STAI-Score (Anxiety)	BDI-Score (Depression)	Audit C-Score (Alcohol Intake)	Igsd9-sf-Score (Videogaming)
**Mean Value (SD)**						
Normal Users	35.47 (14.1)	4.25 (3.0)	23.66 (5.6)	21.20 (2.5)	5.20 (2.0)	11.88 (5.1)
Users with Difficulties	27.43 (9.1) ##	6.58 (3.7) ##	27.16 (7.6) ##	21.20 (2.7)	5.54 (2.2) #	21.51 (9.1) ##
**Bivariated Correlations with MPPUS score**						
Rho (Spearman)	−0.423 **	0.496 **	0.167 **	−0.044 *	0.047 *	0.501 **
Signifficance	*p* < 0.001	*p* < 0.001	*p* < 0.001	*p* < 0.047	*p* < 0.035	*p* < 0.001

(#) Different with respect to Normal Users (*p* ≤ 0.01)/(##) Different with respect to Normal Users (*p* ≤ 0.001), two-tailed. (*) Significant correlation (*p* ≤ 0.05)/(**) Significant correlation (*p* ≤ 0.01), two-tailed. SD, Standard Deviation.

**Table 12:** Binary logistic regression of factors affecting problem phone use (users with difficulties).

MODEL	VARIABLE	B	SEM	W	df	*p* Value	Exp (B)	95% CI for Exp (B)	
								Lower	Upper
**MODEL A** **(All participants)**	Age	−0.050	0.007	58.47	1	0.001	0.95	0.939	0.964
	Hours of Use	0.143	0.018	61.39	1	0.001	1.15	1.113	1.196
	STAI Score	0.091	0.011	72.65	1	0.001	1.09	1.073	1.118
	Audit C Score	0.086	0.030	8.31	1	0.004	1.09	1.028	1.155
**MODEL B** **(Only Videogamers)**	Age	−0.047	0.017	7.61	1	0.006	0.95	0.922	0.986
	Hours of Use	0.108	0.040	7.20	1	0.007	1.11	1.029	1.205
	STAI Score	0.113	0.025	21.16	1	0.001	1.12	1.067	1.174
	Sex	0.684	0.318	4.64	1	0.031	1.98	1.063	3.697
	IGDS9-SF Score	0.139	0.019	50.83	1	0.001	1.15	1.106	1.194

## Data Availability

Data availability is granted upon request.

## Supplementary Materials

Supplementary supporting information can be downloaded at: https://www.mdpi.com/article/10.3390/bs16010008/s1. It contains: Supplementary Table S1. Prevalence of problematic use by education level, based on the MMPUS; Supplementary Table S2. Prevalence of problematic use by main occupation, based on the MPPUS; Document S1. Sociodemographic and Mobile Phone Use Questionnaire.
