# Supplementary material for: Evolution of Problematic Mobile Phone Use in the Spanish Population over the Last Decade
Source: Behav Sci (Basel). 2025 Dec 19;16(1):8. doi: 10.3390/bs16010008 (PMC12838345; doi:10.3390/bs16010008)
Supplement: Supplementary file 1 [file behavsci-16-00008-s001.zip › behavsci-4002482-supplementary.pdf]

SUPPLEMENTARY MATERIALS

Evolution of Problematic Mobile Phone Use in the Spanish Population Over the Last Decade

Jose de-Sola [1,2], Joan Mestre [3] , Victor Villanueva [4], Hernán Talledo [5], Antonia Serrano [1], Gabriel Rubio [6] and Fernando Rodríguez de Fonseca[1*]

Supplementary Table S1 - Prevalence of problematic use by education level, based on the MMPUS

	Without Education	Primary/Basic Education	Middle school, high school	Higher education, university	Total
**Casual Users**					
**2018**	22.2%	19.8%	17.5%	12.4%	15.1%
**2024**	7.7%	12%	12.4%*	7.9	9.6%
**Regular Users**					
**2018**	44.4%*	54.8%.*	65.2%	67.2%	65.3%
**2024**	53.8%	68.3%	69.1%	73.4%	71.5%
**At-risk users**					
**2018**	33.3%*	22.2% *	13.2%	14.8%	14.8%
**2024**	30.8%	14.1%	15.2%	15.8%	15.6%
**Problematic Users**					
**2018**	0.0%	3.2 %	4.0%	5.6%	4.8%
**2024**	7.7%	5.6%	3.3%	2.9%	3.2%
**Users with Difficulties**					
**2018**	33%	28.2%*	17.2%	20.4%	19.6%
**2024**	38.5%	19.7%	18.5%	18.7%	18.8%
**Total**					
**2018**	9	126	644	833	1612
**2024**	13	142	645	1201	2001

(*) Significant percentage difference ($p \geq 0.05$) with respect to the total/(**) Significant percentage difference ($p \leq 0.01$) with respect to the total.

Supplementary Table S2. Prevalence of problematic use by main occupation, based on the MPPUS

		Without specific pursuits or unemployed	Housework	Studying	Working	Total
Casual users						
	**2018**	28.8% **	13.9%	2.8% **	14.2%	15.1%
	**2024**	18.9%**	5.5%*	2.7%**	10.6%	9.6%
Regular users						
	**2018**	64.0%	69.7%	69.3%	64.3%	65.3%
	**2024**	69.74%	72.7%	68.5%	72.9%	71.5%
At-risk users						
	**2018**	5.2% **	12.3%	24.7% **	15.6%	14.8%
	**2024**	8.8%*	18.2%	23.7%*	13.8%	15.6%
Problematic users						
	**2018**	1.9% *	4.1%	3.3%	6.0%	4.8%
	**2024**	2.5%	3.6%	5.1%	2.7%	3.2%
Users with difficulties						
	**2018**	**7.1% ****	**16.4%**	**27.9% ****	**21.5%**	**19.6%**
	**2024**	**11.3%***	**21.8%**	**28.8%****	**16.5%**	**18.8%**
**Total**						
	**2018**	**267**	**122**	**215**	**1008**	**1612**
	**2024**	**238**	**55**	**451**	**1257**	**2001**

(*) Significant percentage difference (p ≥ 0.05) with respect to the total/(**) Significant percentage difference (p ≤ 0.01) with respect to the total.

SOCIODEMOGRAPHIC AND MOBILE PHONE USE QUESTIONNAIRE

1. SOCIODEMOGRAPHIC DATA (5 ITEMS)

1. In which town/city and province of Spain do you usually live?
Town / Province

2. Please write your age (indicate the number of years).
____ years

3. And are you male or female? (Only one answer)
Female 0
Male 1

4. What is your current level of education at the time of this interview? (Only one answer)
Higher education, university 3
Secondary education, baccalaureate 2
Basic/primary education 1
No studies 0

5. Which of the following situations best describes your current main activity? Although you may do several things, indicate your MAIN activity, the one you spend most of your time on each day (Only one answer).
Working 4
Studying 3
Housework 2
Retired 1
Unemployed or without a specific activity 0

2. PERCEPTION, HABITS AND USE OF THE MOBILE PHONE, INCLUDING APPLICATIONS (10 ITEMS)

Now speaking specifically about the mobile phone, the following questions refer only and exclusively to your PERSONAL use of the mobile phone, not for work-related reasons or obligations.

1. How frequently do you think you use your mobile phone? (Only one answer)
A lot 4
Quite a lot 3
Normal, as needed 2
Little, occasionally 1
Rarely, hardly at all 0

2. And honestly, do you think you abuse or are even "hooked" on your mobile phone, as many people say happens to them? (Only one answer)
Definitely yes 4
Probably yes 3
Not very sure 2
Probably not 1
Definitely not 0

3. Approximately how many hours per day do you spend on your mobile phone? (Indicate number of hours.)
____ (hours)

4. And specifically, what do you use your mobile phone for the most? Although you may use it for many things in general, indicate the MAIN uses you most frequently give to your phone. Select all that apply.
To talk 1
To make video calls (Skype, Facetime, etc.) 2
To browse the Internet 3
To chat with friends, family or acquaintances 4
To send or receive emails 5
To send or receive SMS 6
To use and navigate social networks 7
To play video games or similar apps 8
To take photos or videos 9
To listen to music 10
To read the news or press 11
As support when doing sports 12
To connect with the bank 13
To pay, like a credit card 14
To check the weather 15
To place online bets 16
To watch series or movies 17
To entertain myself or pass the time 18
To shop online 19
To listen to radio or watch TV 20

As a GPS, for orientation or geolocation 21
As an agenda, to save appointments and contacts 22
To read books 23
Other uses 24

5. In general, of all the times you pick up or use your mobile phone on a normal day, how often do you do it without any specific purpose, only to entertain yourself, kill time or distract yourself when you are bored and don't know what to do? Please indicate the approximate percentage on a scale from 0% to 100%.
0% 10% 20% 30% 40% 50% 60% 70% 80% 90% 100%

6. And when you are having a bad day, going through a difficult moment or a rough patch, feeling down or upset, of all the times you pick up or use your phone, how often do you do it to calm yourself, escape for a while, or feel accompanied? Please indicate the approximate percentage on a scale from 0% to 100%.
0% 10% 20% 30% 40% 50% 60% 70% 80% 90% 100%

7. Specifically, which of the following mobile applications do you use most frequently, above the rest? Although you may know and have used many of them at some point, indicate ONLY the ones you use habitually, MOST FREQUENTLY. Select all that apply.
X/Twitter 1
Facebook 2
Instagram 3
WhatsApp 4
YouTube 5
Pinterest 6
LinkedIn 7
Spotify 8
Google Plus 9
Viber 10
Snapchat 11
Tumblr 12
Telegram 13
Line 14
Skype 15
Facetime 16
Tinder, Badoo or similar 17
Xing 18
Yuglo 19
Blogging 20
Weplay 21
Wechat 22
Qzone 23
Minube 24
Digg 25
Care2 26
Vkontakte 27
TikTok 28
Threads 29
Others 30

8. And mainly, what are the reasons or purposes for which you use the applications you selected above? Select all that apply.
To share or interact with others 1
To be seen, to let others know what I do 2
To see others, to know what they do 3
To tell things or stay informed 4
To entertain or distract myself 5
To follow others, seek followers or “likes” 6
To increase contacts or relationships 7
To calm myself or reduce anxiety 8
To avoid feeling alone or because I feel alone 9
To project an image I like 10
To feel recognized or valued by others 11
To flirt or meet new people 12
To follow or engage in activities I enjoy 13
To feel included or integrated with others 14
To avoid loneliness or the feeling of loneliness 15
To exchange opinions, feelings or experiences 16
To express who I am, what I feel or what I want 17
For professional networking 18
To feel better during difficult moments 19
To feel closer to my people or family 20
To stay connected 21
Other purposes 22

9. Do you usually turn off your mobile phone when you go to sleep? (Only one answer)
Always 0
Sometimes 1
Never 2

10. And where exactly do you leave your mobile phone when you go to sleep? (Only one answer)
On the bedside table 2
In the same bedroom where I sleep 1
Somewhere else in the house, not in the bedroom 0
